# Disruption of Ah Receptor Signaling during Mouse Development Leads to Abnormal Cardiac Structure and Function in the Adult

**DOI:** 10.1371/journal.pone.0142440

**Published:** 2015-11-10

**Authors:** Vinicius S. Carreira, Yunxia Fan, Hisaka Kurita, Qin Wang, Chia-I Ko, Mindi Naticchioni, Min Jiang, Sheryl Koch, Xiang Zhang, Jacek Biesiada, Mario Medvedovic, Ying Xia, Jack Rubinstein, Alvaro Puga

**Affiliations:** 1 Department of Environmental Health and Center for Environmental Genetics, University of Cincinnati College of Medicine, Cincinnati, Ohio 45267, United States of America; 2 Department of Internal Medicine, Division of Cardiovascular Health and Disease, University of Cincinnati College of Medicine, Cincinnati, Ohio 45267, United States of America; Rutgers University -New Jersey Medical School, UNITED STATES

## Abstract

The Developmental Origins of Health and Disease (DOHaD) Theory proposes that the environment encountered during fetal life and infancy permanently shapes tissue physiology and homeostasis such that damage resulting from maternal stress, poor nutrition or exposure to environmental agents may be at the heart of adult onset disease. Interference with endogenous developmental functions of the aryl hydrocarbon receptor (AHR), either by gene ablation or by exposure *in utero* to 2,3,7,8-tetrachlorodibenzo-*p*-dioxin (TCDD), a potent AHR ligand, causes structural, molecular and functional cardiac abnormalities and altered heart physiology in mouse embryos. To test if embryonic effects progress into an adult phenotype, we investigated whether *Ahr* ablation or TCDD exposure *in utero* resulted in cardiac abnormalities in adult mice long after removal of the agent. Ten-months old adult *Ahr* ^*-/-*^ and *in utero* TCDD-exposed *Ahr* ^*+/+*^ mice showed sexually dimorphic abnormal cardiovascular phenotypes characterized by echocardiographic findings of hypertrophy, ventricular dilation and increased heart weight, resting heart rate and systolic and mean blood pressure, and decreased exercise tolerance. Underlying these effects, genes in signaling networks related to cardiac hypertrophy and mitochondrial function were differentially expressed. Cardiac dysfunction in mouse embryos resulting from AHR signaling disruption seems to progress into abnormal cardiac structure and function that predispose adults to cardiac disease, but while embryonic dysfunction is equally robust in males and females, the adult abnormalities are more prevalent in females, with the highest severity in *Ahr* ^*-/-*^ females. The findings reported here underscore the conclusion that AHR signaling in the developing heart is one potential target of environmental factors associated with cardiovascular disease.

## Introduction

Congenital heart disease (CHD) is the most common type of birth defect worldwide, encompassing 25–30% of all cases of malformation [1], and a major cause of adult cardiovascular morbidity and insufficiency [2–4]. The precise etiological factors underlying CHD incidence remain largely unknown [5,6]. Mutations in specific genes, such as the homeobox transcription factors that govern the early events of cardiogenesis and heart development, have been associated with CHD and adult cardiovascular disease in humans and mice [7–12]. In contrast, non-Mendelian CHD is multifactorial and most likely the product of genetic and environmental interplay [5]. A recent review of 3,772 CHD cases reported that only 11% of the patients had a genetic diagnosis [6], underscoring that the etiology of the greater portion of CHD cases is undetermined and possibly owing to gene-environment interactions. It is increasingly recognized that gene-environment interactions exert their highest impact during development [13–16], a concept is in agreement with the Developmental Origins of Health and Disease (DOHaD) Theory, which proposes that the environment encountered during conception, fetal life, infancy, and early adulthood permanently changes the organism structure, function and metabolism ultimately shaping the long-term control of tissue physiology and homeostasis [17,18]. Consequently, investigating how environmental insults during fetal life result in congenital and adult onset cardiac disease is crucial to understanding the etiology of the vast majority of CHD cases.

The aryl hydrocarbon (Ah) receptor (AHR) is a ligand-activated transcription factor and a member of the basic-region-helix-loop-helix PER/ARNT/SIM (bHLH-PAS) superfamily [15]. The various members of this family function as environmental sensors [19] and differentially regulate signaling pathways related to development and homeostasis [20]. Xenobiotic environmental ligands of the AHR have been shown to adversely affect the cardiovascular system in experimental models *in vitro* [21,22] and *in vivo* [14,15,23–27]. Specifically, the AHR has been linked to cardiovascular health and disease through an endogenous signaling pathway participating in a complex regulatory target network for cardiogenesis and cardiovascular homeostasis [22,27], with enduring functions in postnatal cardiac physiology, such as cardiac sufficiency, blood pressure regulation [28–30], and cardiovascular pathology [31].

TCDD (2,3,7,8-tetrachlorodibenzo-*p*-dioxin; dioxin) is the prototypical and most potent AHR exogenous ligand [32] that off-competes putative endogenous ligands, diverting the receptor from its physiological functions [33], as recently shown with experiments in which *in utero* exposure to TCDD resulted in decreased nuclear localization of the AHR [27]. In parallel, proteasome-dependent degradation of ligand-activated Ah receptor results in “post-exposure AHR down-regulation”, which, at least temporarily, recapitulates an AHR-null condition [34,35]. As a consequence, the combinatorial product of AHR down-regulation and misappropriation, via constitutive or exogenous ligand-induced AHR disruption, dysregulates several biological processes, such as immune response, growth factor signaling, cell cycle proliferation, differentiation, arrest, and apoptosis [14,36–38], as well as cardiogenesis and cardiovascular homeostasis [14,27]. Specifically, the effect of *in utero* AHR disruption during early life embryonic days (E) E13.5, E15.5, and E18.5 delineated covert cardiac morphological functional effects, accompanied by many dysregulated signaling pathways involved in cardiogenesis, cardiac function, and mitochondrial function [27], building on previous *in vitro* evidence that activation, inhibition, or knockdown of *Ahr* during mouse embryonic stem cell differentiation all significantly inhibit the formation of contractile cardiomyocyte nodes [22].

Taken together, these data suggest that *in vitro* and *in vivo* AHR disruption-related effects have the potential to impair postnatal cardiovascular maturation and function, in analogy to the adult cardiac insufficiency resulting from congenital heart disease in humans. Therefore, to address the hypothesis that an altered adult cardiovascular structure and function can be experimentally linked to gestational disruption of the AHR signaling pathway *in vivo*, we studied the molecular, structural, ultrastructural, functional and pathological cardiac phenotypes of adult naïve *Ahr* knockout mice (*Ahr* ^*-/-*^) and wild-type (*Ahr* ^*+/+*^) adult mice exposed *in utero* to TCDD or vehicle. This experimental paradigm of comparing TCDD-exposed and naïve *Ahr* ^+/+^ to *Ahr* ^-/-^ mice was specifically selected as a follow-up of our previous work [27] to determine if developmental environmental disruption or genetic ablation of the *Ahr* gene, respectively, cause cardiac changes associated with physiological deficits in adult life. We find that abnormalities induced by AHR disruption *in utero* persist long after removal of the inducing agent and have a significant effect on predisposing the adult to cardiac disease. In analogy to human CHD, the congenital heart defects induced by AHR disruption in the mouse embryo may be a cause of cardiac insufficiency in the adult.

## Materials and Methods

### Animals and Treatments

All experiments were conducted using the highest standards of humane care in accordance with the NIH Guide for the Care and Use of Laboratory Animals and were approved by the University of Cincinnati Institutional Animal Care and Use Committee. Age-matched *Ahr* ^+/+^ and *Ahr* ^-/-^ C57BL/6J female mice were mated overnight with *Ahr* ^+/+^ and *Ahr* ^-/-^ C57BL/6J male mice, respectively. Maternal gestational exposure to the prototypical AHR ligand TCDD was performed via oral gavage at key developmental time points as previously described [22,27] and is illustrated in detail in Fig A in S1 File. Dams were treated by oral gavage on embryo days E7.5, E9.5 and E11.5 with either corn oil (vehicle) or with TCDD at doses of 0.1 or 1 μg/kg (hereafter referred to as low-dose and high-dose, respectively), which based on previous determinations are estimated to correspond to 0.034 ng and 0.34 ng, respectively, per embryo [39]. TCDD doses were specifically selected to include environmentally-relevant ranges and are within range of the reported body burdens of populations with known exposure for dioxin and dioxin-like compounds, in the range of 0.1–7 μg TEQ/kg [40]. Mice were weaned on postnatal day (PND)21 and males and females were kept separately in standard housing conditions until the scheduled necropsy at PND 300 = 10 months of age).

### Heart samples

At about 10 months of age, following euthanasia, hearts were immediately harvested, rinsed in PBS, gently blotted to remove excess fluids, and weighed using a high definition scale. When appropriate, organ weights were normalized to body weight. Hearts were longitudinally bisected with one half used for morphological studies (i.e. histological processing) and the other half processed for molecular studies (RNA.seq and western blot analysis) as detailed below.

### Histology and Microscopy

Tissues samples were fixed for 48 hours in freshly-prepared 4% paraformaldehyde at 4°C (Sigma-Aldrich), rinsed in serial ethanol dilutions, and then routinely processed for histology. Routine hematoxylin and eosin (H&E) was performed using standard protocols. Masson’s Trichrome (MT) histochemical staining (ScyTek Laboratories, Inc.) and wheat germ agglutinin (WGA) Texas Red^®^-X Conjugate staining (Life Technologies) were performed according to the manufacturer instructions. Slides were visualized and imaged with an Axio Scope. A1 microscope equipped with an AxioCam ERc5s camera (Carl Zeiss Microscopy). MT analyses were performed on three hearts per sex and experimental condition and MT-positive quantification was performed using the color deconvolution plugin and threshold functions of the ImageJ 1.47h (National Institutes of Health) to determine the percentage of MT positive tissue (% positive index). WGA analyses were performed using the manual contour function of the Zeiss’ Zen software (Zeiss Microscopy) to determine the mean cross-sectional myofiber area, on at least 20 cross-sectioned myofibers in five non-overlapping high power fields (40x objective) of at least three hearts per sex and experimental condition.

### Transmission electron microscopy

Microdissected samples from at least three freshly-harvested adult hearts, at about 10 months of age, per sex and experimental condition were immediately fixed in phosphate-buffered 3% glutaraldehyde for 24 hours and submitted to the Pathology Research Core at Cincinnati Children’s Hospital Medical Center for sample processing and sectioning for electron microscopic examination. Samples were washed three times in 0.1 M cacodylate buffer and post-fixed in 1% osmium tetroxide buffered with cacodylate, pH 7.2, at 4°C for 1 h. After dehydration in serial alcohol and propylene oxide solutions, samples were infiltrated with and embedded in LX112. Thin sections were stained with uranyl acetate and lead citrate. Imaging was performed on a transmission electron microscope (7600; Hitachi). Five non-overlapping ultraphotomicrographs per grid were taken at 8000X, 25000X, and 70000X magnifications and further evaluated using Image J 1.47h (http://imagej.nih.gov/ij/) software.

### Mitochondria quantification

Relative quantification of mitochondria in the adult hearts at about 10 months of age (ratio of mitochondrial DNA [mtDNA] to nuclear DNA [nDNA]) was carried out by real-time PCR as previously described [41]. Gene targets were the nuclear cytochrome P450 *Cyp1a1* and the mitochondrial *Nd5* (nicotinamide adenine dinucleotide dehydrogenase-5) genes. Primers for *Cyp1a1* were: forward: 5'- AGGCTCTTCTCACGCAACTC -3’; reverse: 5'- TAAGCCTGCTCCATCCTCTG -3’. Primers for *Nd5* were: forward 5’- TGGATGATGGTACGGACGAA -3’; reverse 5’- TGCGGTTATAGAGGATTGCTTGT -3’.

### RNA.seq analysis

Freshly-harvested adult hearts, at about 10 months of age, from three mice per sex and experimental condition were microdissected into right atrium, left atrium, and ventricles. Individual samples were stored in 200 μL of RNA-Later (Ambion), immediately frozen in liquid nitrogen, and further kept at -80°C until RNA extraction. Total RNA was extracted with the RNeasy Mini Kit (QIAGEN) with Proteinase K and DNAse steps. Additional methodology details for RNA.seq studies is provided in the Materials and Methods in S1 File. Briefly, all steps of library construction, cluster generation, and HiSeq (Illumina) sequencing were performed with biological triplicate samples by the Genomics, Epigenomics and Sequencing Core (GESC) in the University of Cincinnati. Sequence reads were aligned to the genome by using standard Illumina sequence analysis pipeline, and further analyzed by the Statistical Genomics and Systems Biology core in the University of Cincinnati. Differential gene expression analyses between *Ahr* ^+/+^ and *Ahr* ^-/-^ hearts or between AHR ligand-exposed and naïve *Ahr* ^+/+^ hearts was performed separately for males and females. Statistical analyses were performed to identify differentially expressed genes for each comparison using the negative-binomial model of read counts as implemented in the Bioconductor DESeq package. Significant genes were selected based on a false-discovery rate–adjusted *q*-value < 0.0001. RNA.seq data was further analyzed using Ingenuity Pathway Analysis (IPA; Ingenuity^®^ Systems, http://www.ingenuity.com). Genome-wide RNA.seq data have been submitted to the GEO database with access URL http://www.ncbi.nlm.nih.gov/geo/query/acc.cgi?acc=GSE73787.

### Western Blot Analysis

Three mice per sex and experimental condition were euthanized at approximately 10 months of age and the hearts were removed, rinsed with PBS and rapidly frozen in liquid nitrogen. The preparation of total protein was performed by crushing the frozen hearts and placing the samples into lysis buffer (containing: 5% SDS, 50 mM Tris-HCl, pH 7.4, 250 mM Sucrose, 75 mM Urea, 10 mM DTT, protease inhibitor cocktail (Sigma, St. Louis, MO) and phosphatase inhibitor cocktail (Roche Indianapolis, IN). The samples were homogenized using a glass-Teflon homogenizer. Samples were then heated to 95°C for 2 minutes, centrifuged for 5 minutes at 10,000g and the supernatant was placed into a new tube and frozen at -80°C. Total protein concentrations were determined using the BCA Protein Assay Kit (Pierce). Aliquots of total protein were separated on Novex gels (Life Technologies), transferred to nitrocellulose membrane (Life Technologies) and blocked with 5% milk-TBS-Tween. 20 μg of protein was loaded on 12% gels for the phospholamban antibodies; PLNt (Thermo Scientific), p-PLN S16 (Millipore) and p-PLN T17 (Badrilla). Fifty micrograms of protein was loaded on 4–12% gradient gels for the ryanodine receptor antibodies; RYR2a (Thermo Scientific), p-RYR S2808 (Badrilla) and p-RYR S2814 (gift of Dr. Arnold Schwartz, University of Cincinnati) or on a 10% gel for the Sodium/Calcium exchanger (NCX, Swant) and sarco/endoplasmic reticulum Ca^2+^-ATPase (SERCA2, Thermo Scientific) immunoblots [42]. Protein bands were visualized using Western Lightning reagents (Perkin Elmer) and the FluorChemE (ProteinSimple). The densitometry of the bands was determined using AlphaEase software (ProteinSimple formally Alpha Innotech) and normalized to GAPDH for loading control.

### Echocardiography

To evaluate for functional abnormalities a full echocardiographic study was carried out as previously described [43] on three mice per sex and experimental condition starting at PND 60, and repeated at PND 90, 150, and 270. Briefly, mice were anesthetized with isoflurane (1.5–2%), and images were obtained from a parasternal long axis view between 2 and 10 mm in depth in both M-mode and B-mode. Images were taken using the Vevo 2100 Ultrasound system equipped with a MS400 probe (30-MHz centerline frequency), and post-processed by a blinded investigator at a separate workstation as previously described using the Vevostrain software (Visualsonic, Vevo 2100, v1.1.1 B1455). The B-mode, color and Doppler images were analyzed for valvular function and structural abnormalities, while the M-mode images were post-processed for cardiac functional analysis including ventricular size, ejection fraction and cardiac output. The comparisons of cardiac output among groups were carried out not considering peripheral vascular resistance.

### Blood Pressure

At 10 months of age, blood pressure determinations (systolic and mean) were performed on three mice per sex and experimental condition at PND 270 by the tail-cuff method (Visitech Systems ^®^—Apex, NC/BP-2000 ^®^ Blood Pressure Analysis) according to manufacturer instructions. Briefly, mice were acclimatized to the equipment over the course of one week where no data was collected. In the subsequent week, determinations were carried out every-other-day for three days. For every session, 20 determinations were made. The data collected for each mouse was averaged per session and again per sex and group; the reported mean systolic and mean blood pressure per sex and group is the average of all three sessions.

### Exercise Tolerance

Environmental cardiovascular challenge was performed by using an Accupacer Treadmill (Omnitech Electronics, Inc) and assessed at PND 60, 90, 150 and 270 adapted from previously-described methods [44]. Briefly, mice were acclimatized to the equipment through three sham exercise sessions over the course of two weeks where no data was collected. Each exercise session consisted of a warm up phase of five minutes at 10 m/s speed with no incline, followed by the endurance phase at 24 m/s speed and 6 degree incline for up to 60 minutes or until failure. Failure was defined as standing over the stimulus bars for more than five seconds, whereupon the mouse was removed from the treadmill. Time to failure was recorded individually and averaged per sex and experimental condition.

### Statistical Analyses

Statistical analyses were done at each time point using unpaired two-tailed t-test of *Ahr* ^*-/-*^ or TCDD-exposed *Ahr* ^*+/+*^ mice relative to naïve *Ahr* ^*+/+*^ males or females, respectively.

## Results

### Developmental AHR disruption affects adult cardiovascular function

**Fig 1:**
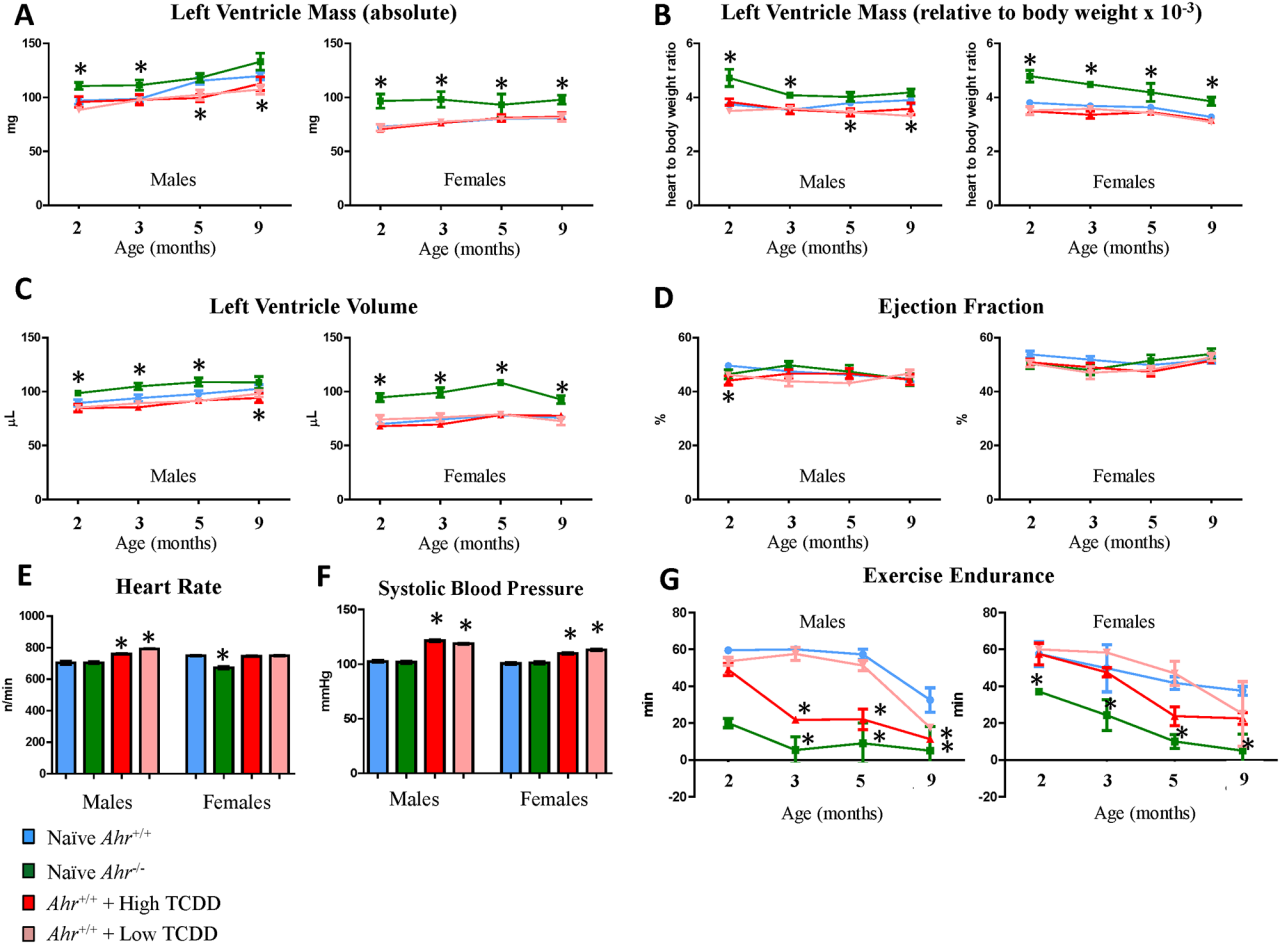
In utero disruption of AHR signaling affects the adult cardiovascular function. Naïve *Ahr* ^+/+^ and *Ahr* ^*-/-*^ and high and low dose TCDD-exposed mice were assessed for cardiovascular function on postnatal days (PND) 60, 90, 150, and 270, corresponding to 2, 3, 5, and 9 months of age, respectively. Absolute left ventricle mass (mg) (A), normalized left ventricle mass (B), left ventricle volume (μL) (C), and ejection fraction (%) (D) were derived from echocardiographic examination of heart function. Conscious heart rate (F) and systolic blood pressure (G) were determined by the tail-cuff method on PND 270. Exercise endurance (minutes) was assessed by forced treadmill exercise on 2, 3, 5, and 9 months of age. All data is expressed as mean ± SEM where *p< 0.05.

To determine whether disruption of AHR signaling *in utero* had long-lasting consequences to heart function and performance in adult mice, we examined echocardiographic endpoints of heart function, blood pressure, and exercise endurance capacity after forced treadmill exercise. Echocardiographic assessments revealed that, relative to naïve *Ahr* ^*+/+*^, *Ahr* ^*-/-*^ males and females had significantly increased left ventricle (LV) mass at 2 and 3 months of age, while *Ahr* ^*-/-*^ females had significantly increased LV mass also at 5 and 9 months of age. Increased LV mass was measurable both in absolute weight (Fig 1A) and in weight normalized to body weight (Fig 1B), while no significant effect was observed to body weight for any of the experimental groups (Fig B in S1 File). Increased LV mass was accompanied by a pronounced increase in LV volume, suggesting the presence of LV dilation (Fig 1C). In contrast, low and high dose TCDD-exposed males had significantly decreased LV mass (both absolute and normalized to body weight) at 5 and 9 months, and decreased LV volume at 9 months. There were no correlative decreases in ejection fraction in any experimental group (Fig 1D). In addition, a significant, albeit slight, increase of the aorta outflow diameter was noted in *Ahr* ^*-/-*^ males and females starting at 5 months and progressing at 9 months (Fig C in S1 File).

In addition, at 10 months of age, males exposed to low- or high-dose TCDD had significantly increased resting (conscious) heart rates, approximately 8% and 13% higher, respectively, than naïve *Ahr* ^*+/+*^ males, while *Ahr* ^*-/-*^ females had a significant 10% decrease in heart rate (Fig 1E). In both males and females, exposure to low- or high-dose TCDD also caused a significant elevation of blood pressure, with increases in the range of 16–18% for males and 10–16% for females, at either TCDD dose and for both systolic (Fig 1F) and mean (Fig D in S1 File) blood pressure.

Exercise endurance, the time lapsed until mice stop running on a forced treadmill exercise protocol, significantly differed between naïve *Ahr* ^*+/+*^ and *Ahr* ^*-/-*^ or TCDD-exposed mice. At all ages, *Ahr* ^*-/-*^ male and female mice significantly underperformed the endurance test in comparison to their *Ahr* ^*+/+*^ counterparts. *Ahr* ^*-/-*^ males lasted 20–30% less than *Ahr* ^*+/+*^ males, with a slight decrement of time at later time points. *Ahr* ^*-/-*^ females lasted 20–65% less than *Ahr* ^*+/+*^ females, with a marked decrement of time at 5 and 7 months. At 3, 5, and 7 months, males exposed to high-dose TCDD lasted 35% less than naïve *Ahr* ^*+/+*^ males. A similar decrement was observed at 7 months in males exposed to low-dose TCDD. Non statistically-significant trends towards decreased endurance time were also noted at 7 months in females exposed to high-dose TCDD (Fig 1G). These results suggest that disruption of AHR signaling *in utero* has long-lasting consequences to cardiovascular function in adult mice, evident when the adult mice are subjected to exercise endurance, an environmental stressor.

### Developmental disruption of AHR signaling alters the structure of the adult myocardium

Exogenous ligand-driven or constitutive AHR signaling disruption during mouse heart development has been associated with molecular, structural, and functional effects to the fetal heart [27] but the extent to which these effects persist or progress in the adult heart has yet to be determined. To that end, we examined the structure and function of *Ahr* ^*-/-*^ and *Ahr* ^*+/+*^ adult mice, the latter exposed *in utero* to TCDD or unexposed.

**Fig 2:**
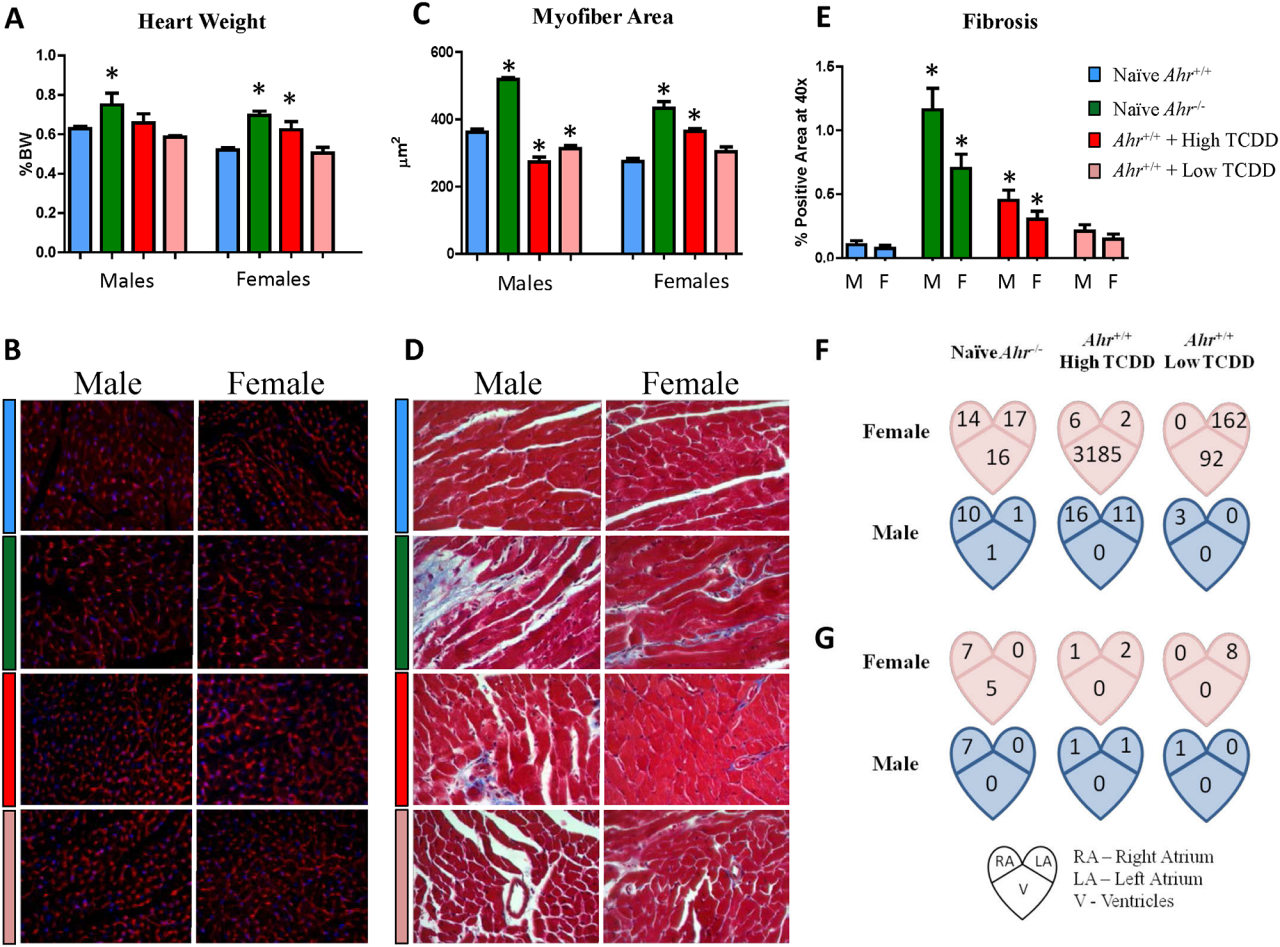
In utero disruption of AHR signaling affects adult heart structure and transcriptome. (A) Heart weights (normalized to body weight) at necropsy (10 months of age, PND 300)) from naïve *Ahr* ^+/+^ and *Ahr* ^*-/-*^ and high and low dose AHR-ligand exposed mice. Wheat germ agglutinin (B) staining of 5-μm sections were used to highlight the cross-sectional myofibers profiles which were used to ascertain the myofiber cross-sectional area (C), which are expressed as means ± minimum and maximal measured areas. Nuclei are stained in blue by DAPI-counterstain; 40X objective. Masson’s trichrome staining (D) was used to highlight the myocardial extracellular matrix as a proxy for fibrosis (E); 40X objective. (F) Number of differentially expressed genes in the adult heart as a consequence of AHR disruption *in utero*, from *Ahr* ^*-/-*^ and TCDD-exposed *Ahr* ^*+/+*^ right atrium (RA), left atrium (LA), and ventricle (V) tissue from males and females. (G) Compartment-specific number of persistent genes (i.e. differentially expressed in the embryo heart and in the adult heart).

At 10 months of age, when compared to naïve *Ahr* ^*+/+*^, *Ahr* ^*-/-*^ males and females showed approximately 19% and 33%, respectively, significant increases in mean heart weight relative to body weight while high-dose TCDD-exposed *Ahr* ^*+/+*^ females had a significant 11% increase in mean heart weight relative to body weight (Fig 2A). To further characterize the altered heart mass, we stained 5 μm-thick sections with fluorescently-labeled wheat germ agglutinin (Fig 2B) to assess the morphometrical quantification of cross-sectional myofiber area. Heart weight increases in *Ahr* ^*-/-*^ males and females correlated with significant increases in mean myofiber cross-sectional area of 44% and 58%, respectively, while *Ahr* ^*+/+*^ females exposed to high-dose TCDD had a 33% significant increase in mean myofiber cross-sectional area (Fig 2C). In contrast, while the overall spread of individual myofiber areas was similar between naïve and TCDD-exposed males, high- and low-dose exposed males showed a significant loss of 24% and 13%, respectively, of myofiber cross-sectional area, correlated to the observed lower heart weights. These findings suggest that the observed increased in heart weights of *Ahr* ^*-/-*^ males and females and high-dose TCDD-exposed *Ahr* ^*+/+*^ females is due to an increase of individual myofiber mass, consistent with cardiomyocyte hypertrophy.

Histological sections were stained with Masson’s trichrome (Fig 2D) to assess myocardial remodeling by highlighting the differences in the amount of interstitial extracellular matrix, which were quantified as a proxy for myocardial fibrosis. *Ahr* ^*-/-*^ males and females had a significant, although small, increase in interstitial fibrosis, from about 0.1% positive sectional area in naïve *Ahr* ^*+/+*^ males and females to about 1.25% and 0.75% in *Ahr* ^*-/-*^ male and female hearts, respectively (Fig 2E). *Ahr* ^*+/+*^ mice exposed to high-dose TCDD had a significant slight increase of about 0.4% and 0.3% in males and females, respectively. Altogether, these data suggest that disruption of AHR signaling *in utero* and its effects on the developing embryonic myocardium that we had previously observed [27], do in fact persist and affect myocardial structure in the adult.

### Disruption of AHR signaling during development has lasting effects in the adult heart transcriptome

A key question in the analysis of the role of gene-environment interactions in the developmental genesis of adult cardiac disease is whether interference with AHR endogenous functions, either by gene ablation or by exposure to TCDD *in utero*, causes persistent abnormalities detectable in both the embryo and the adult. In our previous work, we observed that a significant fraction of the mouse transcriptome, needed for attainment and maintenance of cardiac differentiation, was deregulated with striking similarity by both loss of the *Ahr* gene and exposure to TCDD *in utero* [27]. To search for genes that might be differentially expressed in the adult heart as a consequence of AHR disruption *in utero*, we used deep sequencing of the heart transcriptomes of *Ahr* ^*-/-*^ and TCDD-exposed *Ahr* ^*+/+*,^ including right and left atrium, and ventricle tissue from males and females. Relative to naïve *Ahr* ^*+/+*^ hearts, comparable numbers of genes were differentially-expressed in the right atrium of *Ahr* ^*-/-*^ males and females. The same was the case in *Ahr* ^*+/+*^ males and females that had been exposed to high dose TCDD *in utero*, and in the left atrium and ventricles of *Ahr* ^*-/-*^ females. No significant gene numbers were found in the left atrium and ventricles of *Ahr* ^*-/-*^ males and low-dose TCDD-exposed *Ahr* ^*+/+*^ males, and in the ventricles of high dose AHR ligand-exposed *Ahr* ^*+/+*^ males, or right atrium of low-dose TCDD-exposed *Ahr* ^*+/+*^ females (Fig 2F). Curiously, sexually dimorphic quantitative differences were noted as female hearts tended to have an overall higher number of differentially-expressed genes in all experimental groups.

To determine whether expression of specific genes affected in the embryo in our prior work, either at E13.5, E15.5, or E18.5 [27], persisted in the adult heart at 10 months of age we carried out a comparison of the adult transcriptome to the transcriptome of embryonic hearts following AHR disruption *in utero* (following the design detailed in Fig E in S1 File). The compartment-specific number of persistent genes, *i*.*e*., genes differentially expressed in the embryo heart and also in the adult heart, is shown in Fig 2G while their respective identities are listed in Table A in S1 File. The right atrium of *Ahr* ^*-/-*^ males and females and the left atrium of low dose TCDD-exposed females show the highest number of overlapping genes. Although no particular gene signature was noted, selected individual persistent genes are annotated to cardiovascular ontogenies, such as *Col5a3*, *Nppa*, and *Wdr83*, and mitochondrial function, such as *Cox14* and *Mrpl12*.

**Fig 3:**
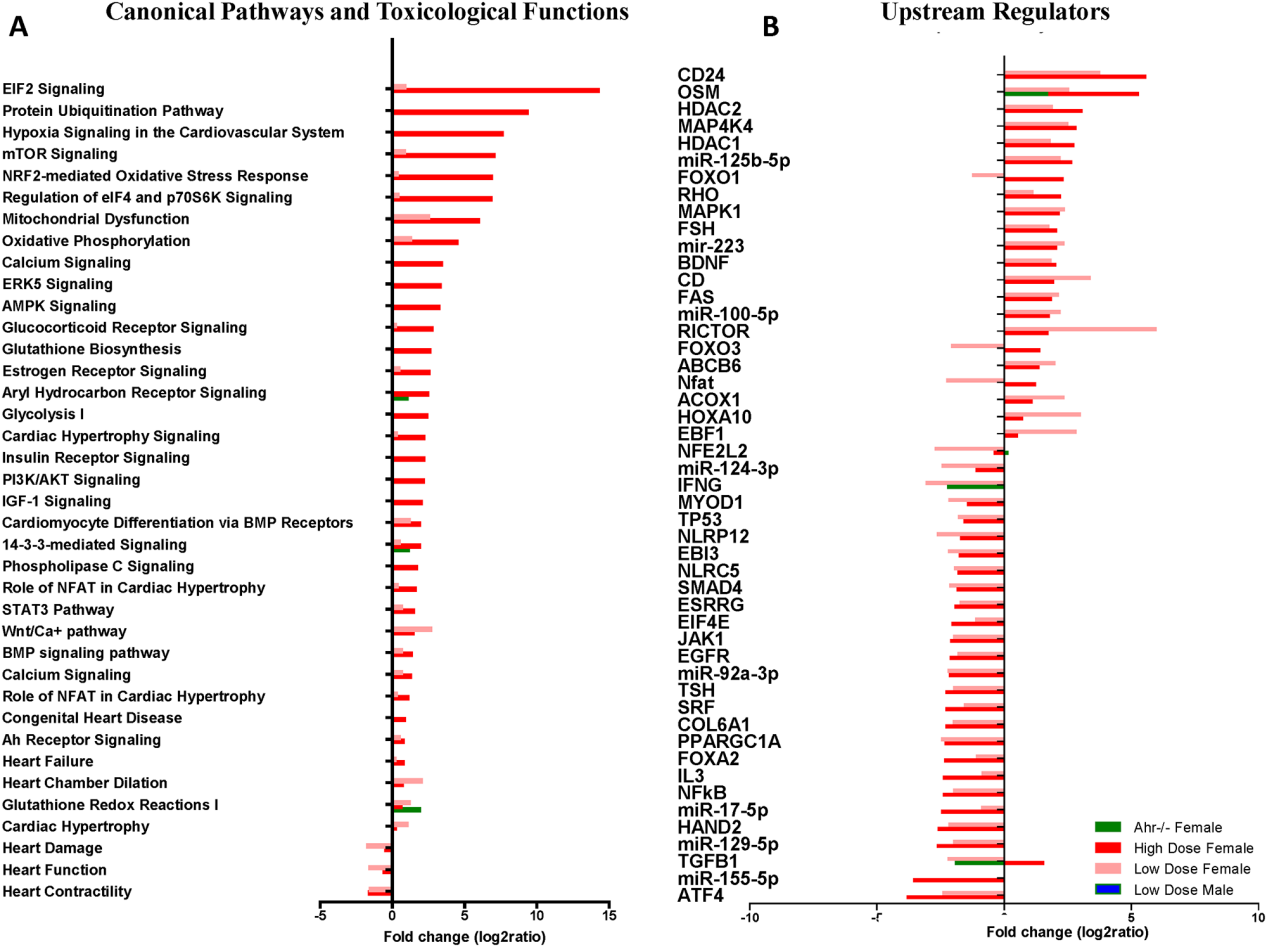
Differentially expressed canonical pathways, toxicological functions, and upstream regulators underlie AHR disruption-driven altered structure and function. (A) Fold change expression of differentially expressed Ingenuity Pathway Analysis (IPA) canonical pathways and toxicological functions from and *Ahr* ^*-/-*^ females and high and low dose TCDD-exposed females relative to naïve *Ahr* ^+/+^. (B) IPA’s upstream regulators from and *Ahr* ^*-/-*^ and high and low dose TCDD-exposed mice relative to naïve *Ahr* ^+/+^.

Comprehensive transcriptome analysis via the Ingenuity Pathway Analysis platform revealed aberrant regulation of genes globally associated with cardiovascular disease together with individual genes associated with mitochondrial function. Genes important for maintaining cardiac structural homeostasis, contractility, promoters of cardiac hypertrophy, and regulating cardiomyocyte energy homeostasis had significantly altered expression in the AHR agonist-exposed female hearts, with an overall higher magnitude of change in high dose TCDD-exposed females (Fig 3A). These changes were generally not observed in *Ahr* ^*-/-*^ hearts or in the hearts of males exposed to TCDD. Among the down-regulated pathways were IPA’s Toxicological Functions annotated to heart damage, function, and contractility. Up-regulated pathways included hypoxia signaling in the cardiovascular system, calcium signaling, mitochondrial dysfunction, oxidative stress response, and hormone nuclear receptor signaling.

**Fig 4:**
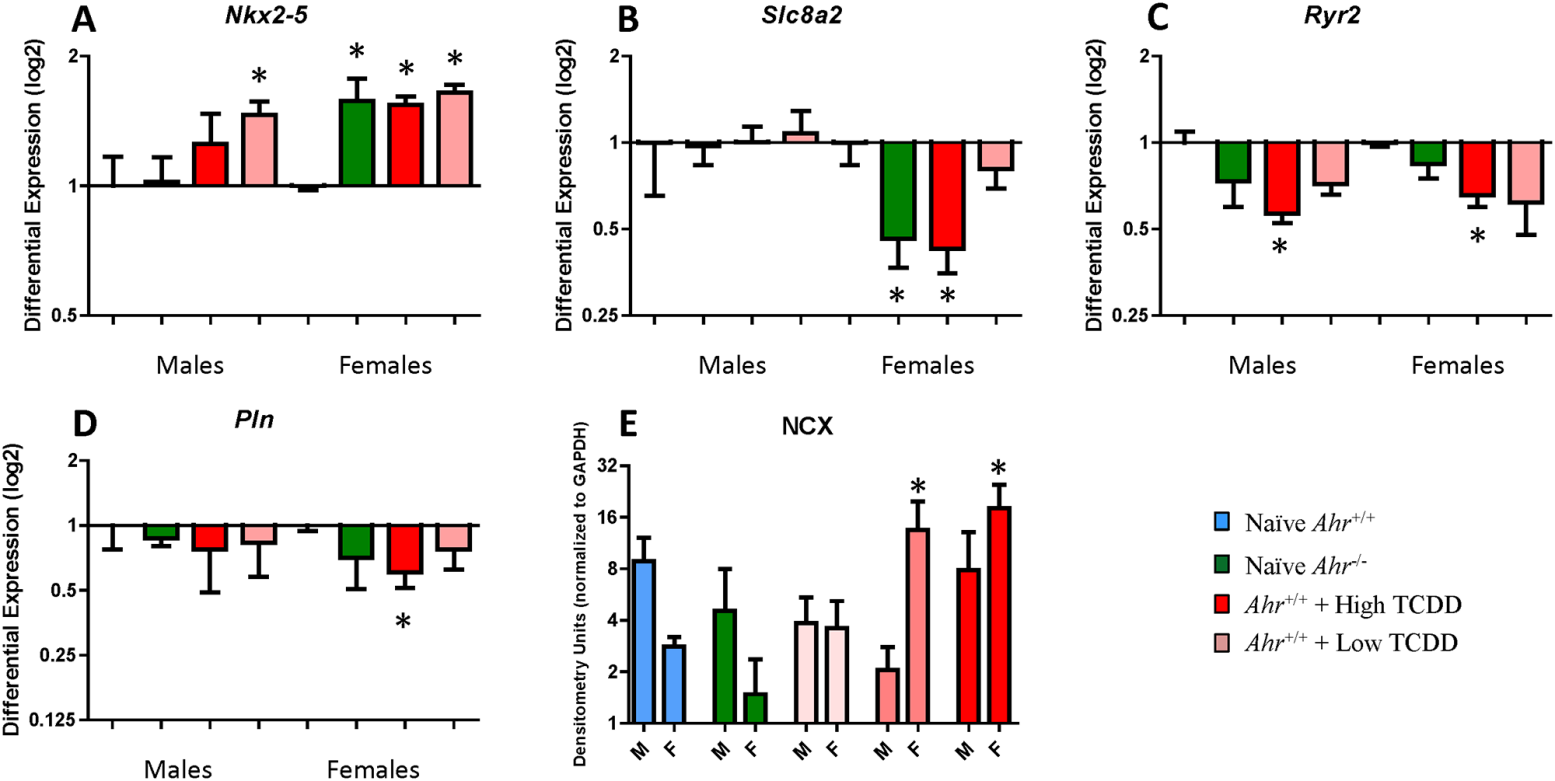
In utero disruption of AHR signaling modestly affects calcium-handling signaling. Fold change mRNA expression of *Nkx2-5* (A), *Slc8a2* (B), *Ryr2* (C), and *Pln* (D), and protein expression of NCX (E) from naïve *Ahr* ^+/+^ and *Ahr* ^*-/-*^ and high and low dose TCDD-exposed mice at 10 months of age (PND 300) (mean ± SEM).

Analysis of upstream regulators identified potential genes predicted to contribute directly or indirectly to the observed aberrant regulation of these genes (Fig 3B). Specific regulators included *Hand2* (Heart and Neural Crest Derivatives Expressed 2) and miR-155. *Hand2* has been identified as a key contributor to congenital heart disease [45] and was 2- to 2.5-fold decreased in expression in high- and low-dose TCDD-exposed female hearts, miR-155, recently associated with hypertrophic cardiomyopathy in humans [46], was decreased about 3-fold in high-dose TCDD-exposed female hearts. Also notably, expression of the cardiogenic transcription factor *Nkx2-5* was significantly increased in the ventricles of *Ahr* ^*-/-*^, and low- and high-dose TCDD-exposed *Ahr* ^*+/+*^ females (Fig 4A) as previously reported in cardiac hypertrophy [47].

In summary, disruption of AHR signaling during development yields lasting effects in the adult heart transcriptome, chiefly on networks related to cardiovascular homeostasis and function, with quantitative and qualitative differences between the atria and ventricles and with a predominance of effects in the adult female heart.

### Canonical calcium-handling mechanisms in the adult heart are preserved following AHR disruption in utero

To investigate whether calcium signaling had a role in the observed alterations of myocardial structure, we followed the expression of a selected panel of calcium regulated genes and proteins involved in cardiac function and homeostasis known to change expression levels and/or phosphorylation status in heart disease, including *Rcan1*, *Pln*, *Ryr2*, *Slc8a2*, *Slc8a3*, *Slc8a4*, *Atp2a1*, and *Atp2a2* [48]. Proteins included phosphorylated phospholamban (p-PLN) and total phospholamban (t-PLN), phosphorylated ryanodine receptor 2A (p-RyR2) and total phosphorylated ryanodine receptor 2A (t-RyR2), sodium-calcium exchanger (NCX), and the cardiac isoform of the SERCA2. A summary of the genes and proteins evaluated is included in Table B in S1 File.

In comparison to wild-type *Ahr* ^*+/+*^ hearts, we only found changes in the expression of *Slc8a2*, *Ryr2*, and *Pln*. *Slc8a2* mRNA levels showed a significant decrease in the ventricles of *Ahr* ^*-/-*^ females and *Ahr* ^*+/+*^ females exposed to high-dose TCDD (Fig 4B). Expression of *Ryr2* was lower in the left atrium and ventricles of *Ahr* ^*+/+*^ males and females exposed to high-dose TCDD and in the left atrium of *Ahr* ^*-/-*^ females and *Ahr* ^*+/+*^ females exposed to low-dose TCDD (Fig 4C). Expression of *Pln* was lower in the ventricles of *Ahr* ^*+/+*^ females exposed to high-dose TCDD (Fig 4D). With regard to changes in protein levels, western blot analysis showed changes only in the levels of the sodium-calcium exchanger NCX, which increased expression by 9- and 6-fold in *Ahr* ^*+/+*^ females exposed to both high- and low-dose TCDD, respectively (Fig 4E). All remaining trends in protein levels or phosphorylation status between naïve *Ahr* ^*+/+*^ mice and other genotype or treatment conditions showed no significant differences (data not shown). The observed differentially expressed genes and protein data suggest that canonical calcium handling protein networks are largely preserved and, therefore, are not major contributors to the altered myocardial structure driven by AHR activation or loss-of-function.

### Mitochondrial homeostasis in the adult heart is affected by developmental AHR disruption

**Fig 5:**
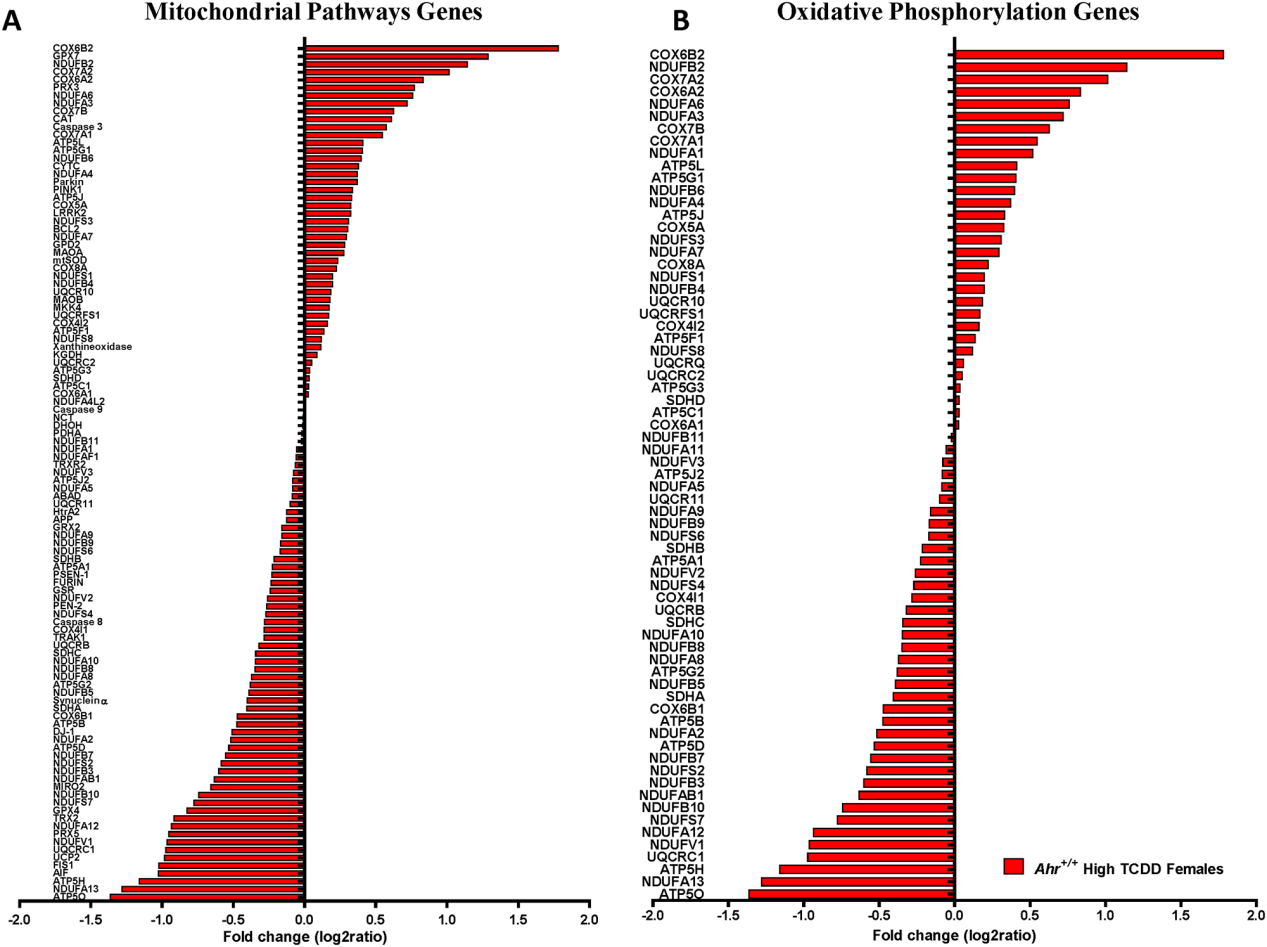
Mitochondrial dysfunction and altered oxidative phosphorylation genes in the adult heart as a result of in utero disruption of AHR signaling. Fold change of expression of significantly altered genes in (A) functional mitochondrial pathways and (B) oxidative phosphorylation (B) at 10 months of age (PND 300) of high dose TCDD-exposed *Ahr* ^+/+^ female adult hearts.

Mitochondrial dysfunction has been associated with AHR signaling disruption *in vitro* [49,50] and *in vivo* [27,51–54], and extensively investigated as a key player in cardiovascular disease [55–57]. Of particular interest to this study, mitochondrial dysfunction with compensatory increased mitochondrial abundance was observed in embryonic *Ahr* ^*-/-*^ or TCDD-exposed *Ahr* ^*+/+*^ embryonic hearts [27]. In agreement with these findings in embryonic hearts, ventricles of adult females exposed *in utero* to high-dose TCDD showed highly significant changes in the expression of genes in the canonical mitochondrial pathway (Fig 5A) and in the oxidative phosphorylation pathway (Fig 5B), relative to naïve *Ahr* ^*+/+*^ females. A small subset of changes was concordant between low-and high-dose TCDD. No similar findings were noted in *Ahr* ^*-/-*^ females or in any of the males.

**Fig 6:**
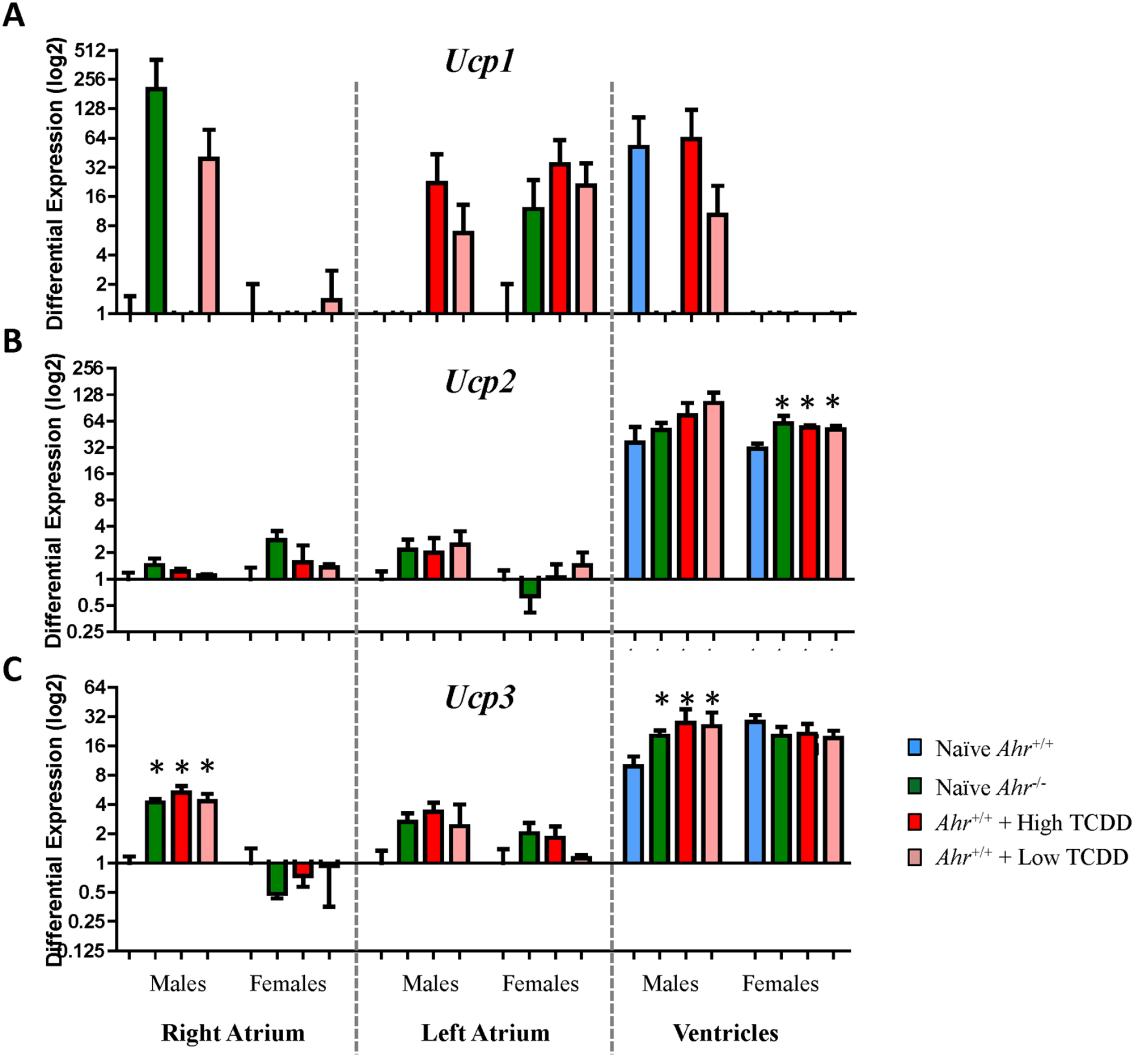
Uncoupling proteins are differentially expressed following in utero disruption of AHR signaling. Fold change of expression of *Ucp1* (A), *Ucp2* (B), and *Ucp3* (C) at 10 months of age (PND 300) of naïve *Ahr* ^+/+^ and *Ahr* ^*-/-*^ and high and low dose TCDD-exposed mice.

Changes in two of the mitochondrial pathway genes, *Cox14* and *Mrpl12*, which we found to be dysregulated in embryo hearts as a result of AHR disruption [27] persisted in the adult hearts. *Cox14* (Cytochrome C Oxidase Assembly Homolog 14), encodes a protein central to the synthesis and assembly of Complex IV and has been associated with mitochondrial Complex IV deficiency [58]. *Mrpl12* (Mitochondrial Ribosomal Protein L12) encodes a nuclear-encoded mitochondrial ribosomal protein essential for mitochondrial protein synthesis [59] that has been associated with disturbed mitochondrial function and dilated cardiomyopathy [60]. In addition, members of the UCP uncoupling protein family were also among the dysregulated mitochondrial function genes. Gene expression levels of the three members, *Ucp1*, *Ucp2*, and *Ucp3*, were measured by qRT-PCR in atria and ventricles of all experimental males and females. No significant differences or trends were noted for *Ucp1* (Fig 6A). *Ucp2* mRNA levels were significantly increased in the ventricles of *Ahr* ^*-/-*^ females and in *Ahr* ^*+/+*^ females exposed to either dose of TCDD (Fig 6B). *Ucp3* mRNA levels were about 4- to 5-fold significantly increased in the right atrium of *Ahr* ^*-/-*^ and high and low dose TCDD-exposed males, respectively; a similar albeit not statistically-significant trend was noted in the left atrium of *Ahr* ^*-/-*^ and high and low dose TCDD-exposed males. In the ventricles, *Ucp3* mRNA levels were about 2- to 3-fold significantly increased in *Ahr* ^*-/-*^ and high and low dose TCDD-exposed males (Fig 6C).

**Fig 7:**
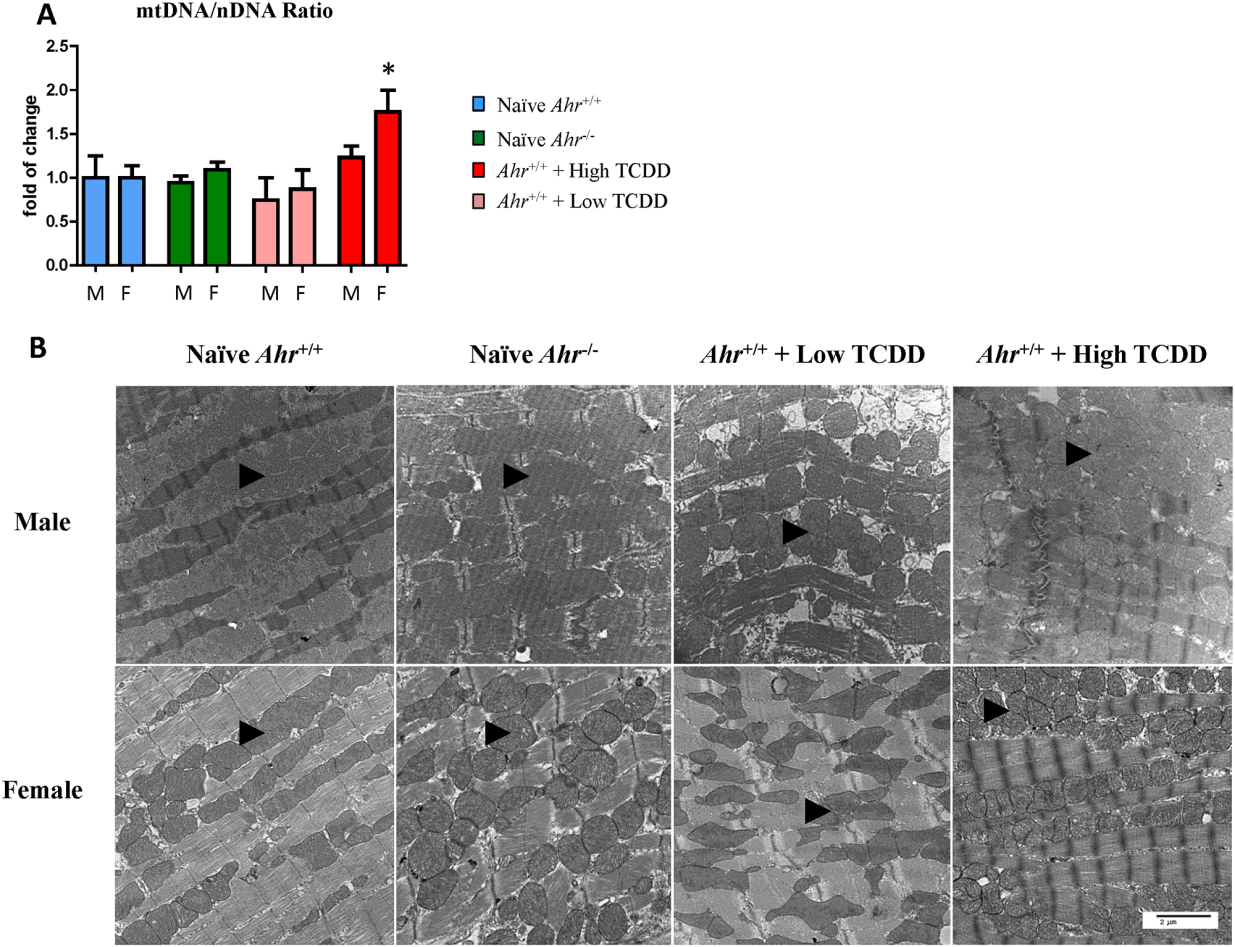
Disruption of AHR signaling induces changes in mitochondria abundance and structure. (A) Quantification of heart mitochondrial DNA relative to nuclear DNA, defined as the ratio of mtDNA to nDNA ratio at 10 months of age (PND 300) and expressed as the mean fold change relative to naïve *Ahr* ^*+/+*^ hearts ± SEM; * p≤0.05. (B) Ultra-thin sections of embryonic hearts collected at 10 months of age from *Ahr* ^*-/-*^ and *Ahr* ^*+/+*^, either naïve or exposed to high or low dose ligand *in utero*, were used for transmission electron microscopy evaluation of the embryonic heart ultrastructure. Illustrative photomicrographs showing the density of mitochondria (*arrowheads*) within the cardiomyocyte sarcoplasm in *Ahr* ^*-/-*^ and in high and low dose TCDD-exposed *Ahr* ^*+/+*^ mice compared to naïve *Ahr* ^*+/+*^. Uranyl acetate and lead citrate stain. Scale bar = 2 μm.

Hearts from *Ahr* ^*-/-*^ and high- and low-dose TCDD-exposed *Ahr* ^*+/+*^ mice were examined for quantity and quality of heart mitochondria. A relative quantification of mitochondria, as determined from the ratio of mtDNA to nuclear DNA, revealed that hearts from females exposed *in utero* to high-dose TCDD had a statistically significant 1.75-fold higher mitochondrial abundance relative to naïve *Ahr* ^*+/+*^ females (Fig 7A). No other comparison showed statistical significant, even though exposed wild-type males showed a trend of an increase over control that did not reach significance (Fig 7A). Ultrastructurally, although no alterations to individual mitochondria morphology were noticed, the increase in relative mitochondria numbers correlated with a slightly higher sarcoplasm density in ventricular cardiomyocytes of females gestationally-exposed to high-dose TCDD (Fig 7B).

In sum, developmental AHR signaling disruption *in vivo* results in altered mitochondrial homeostasis as characterized by aberrant expression of genes belonging to mitochondrial function and oxidative phosphorylation, and correlative increase in relative and ultrastructural mitochondrial abundance in females gestationally-exposed to high-dose TCDD.

## Discussion

To gain further insight into the role of the Ah receptor in the potential gene-environment interactions underlying adult cardiovascular health, we induced AHR disruption, by genetic ablation or by ligand-induced down-regulation, during *in utero* cardiogenesis [27] and followed the mice into adulthood. We find that developmental interference with endogenous AHR functions causes molecular, structural, and functional departures from homeostasis in the adult hearts; some of the alterations are reminiscent of the alterations observed under similar conditions in the embryonic mouse heart [27]. However, unlike the embryonic hearts, which showed extensive similarities between *Ahr* genetic ablation and ligand-induced down-regulation, the adult hearts from the same mice showed a limited number of similarities and this were more evident in the female adult *Ahr* ^*-/-*^ mice than in the males. A summary of these observations is shown in Table C in S1 File.

At 10 months of age, adult *Ahr* ^*-/-*^ male and female mice had pronounced hypertrophic cardiomyopathy evidenced by increased heart weight, ventricular mass myofiber cross-sectional area, interstitial fibrosis, and diastolic left ventricle volume. *Ahr* ^*-/-*^ males had slightly decreased resting heart rates and there were no significant blood pressure differences in *Ahr* ^*-/-*^ males or females. Even though the magnitude of change of interstitial fibrosis is not likely to be a major contributor to the observed increased in heart weights, it is evidence of ongoing myocardial remodeling with loss of contractile myofiber units and replacement with non-contractile and non-conducting supporting matrix. While the heart function in *Ahr* ^*-/-*^ mice was largely compensated at resting conditions, as judged from the lack of correlative decreases in ejection fraction, there was a major effect exercise endurance under strenuous forced running. Overall, these findings are in agreement with previous findings in *Ahr* knockout mice [30], although the progressive left ventricular dilation and the higher severity in *Ahr* ^*-/-*^ females specifically have not been previously reported. Sex- and ethnicity-dependent dimorphic differences in the prevalence of cardiac disease have been epidemiologically attributed to social and socio-economic reasons [61] and differences in the outcomes of cardiac disease complications have been reported [4].

At 10 months of age, males exposed to high-dose TCDD had a milder cardiac phenotype characterized by slightly decreased left ventricle mass from PND150 onwards, and decreased diastolic left ventricle volume at PND270. These findings generally correlated with a larger proportion of cardiac myofibers having smaller cross-sectional area, corroborating that AHR disruption through exposure to a potent ligand like TCDD results in decreased myocardial mass, as described previously [16,27,62]. High- and low-dose exposure to TCDD in males and females also had significantly increased systolic and mean blood pressure at PND 270, and the males had increased resting heart rates. AHR-mediated increases in systemic blood pressure have been previously documented [63], although this study may represent the first correlation between *in utero* AHR disruption and altered blood pressure in the adult. In and of itself, chronic hypertension is a highly prevalent and one of the most powerful contributors to cardiovascular disease in humans [64,65].

In spite of the absence of overt echocardiographic functional alterations, males and females exposed to high-dose TCDD also displayed decreased exercise endurance under strenuous forced running, similar to what we noted in *Ahr* ^*-/-*^ mice. Exercise endurance is a complex phenotype determined by the collective contributions of the central nervous system, cardiovascular and respiratory systems, and skeletal muscle, as well as systemic metabolism [44], where the cardiovascular system and energy metabolism critically contribute to positive exercise outcomes. Specifically, tissue perfusion, a direct consequence of preserved cardiovascular homeostasis, and mitochondrial capacity are tightly correlated with positive exercise outcomes [66]. Our observations suggest that AHR disruption impairs exercise endurance via mechanisms which include, but are not limited to, the cardiovascular system and energy metabolism. A more focused analysis must await data from heart-specific *Ahr* knockout mice. In agreement with this notion, genes important to maintain cardiac structural homeostasis, contractility, promotion of cardiac hypertrophy, and regulation of cardiomyocyte energy homeostasis had significantly altered levels of expression in the TCDD-exposed female hearts, but not in *Ahr* ^*-/-*^ hearts or in TCDD-exposed males. Down-regulated pathways were annotated to heart damage, function, and contractility, while up-regulated pathways included hypoxia signaling in the cardiovascular system, calcium signaling, mitochondrial dysfunction, oxidative stress response, and hormone nuclear receptor signaling. Upstream regulators predicted to explain these effects included critical players such as *Hand2* and miR-155, which have been associated with congenital heart disease [67] and hypertrophic cardiomyopathy [46], respectively.

Surrogate calcium handling protein networks contributed modestly to the AHR-disruption driven altered myocardial structure in *Ahr* ^*-/-*^ and TCDD-exposed mice with only slightly altered expression of key calcium-handling networks noted at the mRNA (decreased *Slc8a2*, *Ryr2*, and *Pln*) and protein (increased NCX) levels. Increased NCX levels have been linked to the genetic reprogramming that underlies cardiac remodeling and hypertrophy [68]. When compared to the transcriptome of embryonic hearts after AHR signaling disruption *in utero* [27], the adult transcriptome bears no striking signature of persistent genes, though expression of some genes, such as *Col5a3*, *Nppa*, *Cox14*, *Mrpl12*, and *Wdr83*, persisted in some experimental groups and are annotated to cardiovascular ontogenies or mitochondrial function.

Mitochondrial function and oxidative phosphorylation pathways were significantly down-regulated in TCDD-exposed *Ahr* ^*+/+*^ female but not male hearts, nor in *Ahr* ^*-/-*^ mice of either sex, once more illustrating a more pronounced phenotype in females. Sex-related differences have been observed in cardiovascular diseases in humans and dimorphic gene expression has been recently observed in the expression of mitochondrial genes in young and old rats [69], although information regarding differential mitochondrial activity and development of cardiac diseases between the sexes is lacking in both humans and laboratory animals. Dimorphism is unlikely to result from a single unifying mechanistic model, but rather from the integration of multiple mechanisms involving hormonal regulation of gene expression, associated with estrogen levels.

Members of the mitochondrial uncoupling family (UCP), Ucp2 and Ucp3, were up-regulated in one or more heart compartments of *Ahr* ^*-/-*^ and high- and low-dose TCDD-exposed mice, which suggests a compensatory response, as UCP2 is typically decreased in expression in the failing heart [70]. These findings are in line with previous studies showing that AHR disruption is associated with mitochondrial dysfunction [27,50] and with UCP dysregulation [71]. The observed molecular alterations to mitochondrial function and oxidative phosphorylation were accompanied by a slight increase in mitochondria abundance, similar to previous observations in embryos [27], limited to high-dose TCDD-exposed female hearts and without accompanying mitochondrial ultrastructural abnormalities.

## Conclusions

The immediate effects triggered in the embryo by the disruption of endogenous AHR functions do not seem to subside with age but rather diverge and diversify into multiple directions encompassing pathways controlling cardiomyocyte structure and function, including hypertrophy signaling and mitochondrial function, ultimately resulting in compensated adult cardiomyopathy. Multiple interconnected compensatory responses are likely at play in the adult heart to cope with the developmental setbacks resulting from AHR-disruption. Seemingly, constitutive ablation of the AHR yields sustained and more complex molecular departures from homeostasis, relative to pharmacological disruption of the receptor, which ultimately translates into a more severe adult phenotype. In addition, maternal cardiovascular dysfunction and abnormal placental development may contribute to the more pronounced phenotype in *Ah*r^-/-^ mice. Interestingly, across all endpoints evaluated, a majority of the findings driven by AHR disruption described in this study are generally more pronounced in females than in males, underscoring the sexual dimorphism of the phenotypes. Unlike in the embryonic heart, where AHR ablation and dowregulation by ligand appear to follow parallel pathways, these two conditions are substantially more divergent in the adult heart. While no overt cardiac insufficiency was observed at 10 months of age, our findings suggest that the compensatory mechanisms at play may be insufficient to cope with added environmental stressors. Our findings extend our understanding of the central role of the AHR signaling network in cardiovascular function and dysfunction, which makes it an important gene-environment nexus in environmental cardiac injury.

## Supporting Information

S1 File Materials and Methods. **Figs A-E**. (A) Experimental design for gestational exposure to the prototypical AHR ligand (TCDD). (B) Body weight (grams) from naïve *Ahr* ^*+/+*^, low- and high-dose ligand-exposed *Ahr* ^*+/+*^, and *Ahr* ^*-/-*^ males and females at the indicated age (in days). Mean ± SEM. (C) Echocardiographic assessment of aorta diameter (μm) from naïve *Ahr* ^*+/+*^, ligand-exposed *Ahr* ^*+/+*^, and *Ahr* ^*-/-*^ males and females at the indicated age (in days). Mean ± SEM; * p≤0.05. (D) Mean blood pressure (mmHg) from naïve *Ahr* ^*+/+*^, ligand-exposed *Ahr* ^*+/+*^, and *Ahr* ^*-/-*^ males and females at 9 months (post natal day [PND] 270). Mean ± SEM; * p≤0.05. (E) RNA.seq data analysis design for adult heart transcriptome at PND 300 and comparison relative to embryo heart (i.e., persistent genes) transcriptome at either E13.5, E15.5, or E18.5. **Table A**: Identity of genes differentially expressed in the embryo at either E13.5, E15.5, or E18.5 which persisted in the adult hearts at post natal day (PND) 300. **Table B**: Identity, function, targets, and fate of selected cardiovascularsignaling pathways genes and proteins in cardiac hypertrophy and failure. **Table C**: Summary of cardiovascular findings related to developmental *Ahr* disruption in male and female mice. Arrow up = upregulated/increased, arrow down = downregulated/decreased, “↔” = not affected.
(DOCX)
