# Supplementary material for: Disruption of Ah Receptor Signaling during Mouse Development Leads to Abnormal Cardiac Structure and Function in the Adult
Source: PLoS One. 2015 Nov 10;10(11):e0142440. doi: 10.1371/journal.pone.0142440 (PMC4640841; doi:10.1371/journal.pone.0142440)
Supplement: S1 File — Figs A-E. (A) Experimental design for gestational exposure to the prototypical AHR ligand (TCDD). (B) Body weight (grams) from naïve Ahr +/+, low- and high-dose ligand-exposed Ahr +/+, and Ahr -/- males and females at the indicated age (in days). Mean ± SEM. (C) Echocardiographic assessment of aorta diameter (μm) from naïve Ahr +/+, ligand-exposed Ahr +/+, and Ahr -/- males and females at the indicated age (in days). Mean ± SEM; * p≤0.05. (D) Mean blood pressure (mmHg) from naïve Ahr +/+, ligand-exposed Ahr +/+, and Ahr -/- males and females at 9 months (post natal day [PND] 270). Mean ± SEM; * p≤0.05. (E) RNA.seq data analysis design for adult heart transcriptome at PND 300 and comparison relative to embryo heart (i.e., persistent genes) transcriptome at either E13.5, E15.5, or E18.5. Table A: Identity of genes differentially expressed in the embryo at either E13.5, E15.5, or E18.5 which persisted in the adult hearts at post natal day (PND) 300. Table B: Identity, function, targets, and fate of selected cardiovascularsignaling pathways genes and proteins in cardiac hypertrophy and failure. Table C: Summary of cardiovascular findings related to developmental Ahr disruption in male and female mice. Arrow up = upregulated/increased, arrow down = downregulated/decreased, “↔” = not affected. (DOCX) [file pone.0142440.s001.docx]

**Disruption of Ah Receptor Signaling during Mouse Development Leads to Abnormal Cardiac Structure and Function in the Adult**

Vinicius S. Carreira^1^, Yunxia Fan^1^, Hisaka Kurita^1^, Qing Wang^1^, Chia-I Ko^1^, Mindi Naticchioni^2^, Min Jiang^2^, Sheryl Koch^2^, Xiang Zhang^1^, Jacek Biesiada^1^, Mario Medvedovic^1^, Ying Xia^1^, Jack Rubinstein^2^, and Alvaro Puga^1,^ ^*^

^1^Department of Environmental Health and Center for Environmental Genetics

University of Cincinnati College of Medicine, Cincinnati, Ohio 45267, USA^2^Department of Internal Medicine, Division of Cardiovascular Health and Disease

University of Cincinnati College of Medicine, Cincinnati, Ohio 45267, USA

**Supplemental File S1 - List of Contents**

**Supplemental Materials and Methods**

- 1. Animals and Treatments
  2. RNA.seq analysis

**Supplemental Figures**

- 1. Figures A-E

**Supplemental Tables**

- 1. Table A
  2. Table B
  3. Table C

**Materials and Methods**

1. **Animals and Treatments**

All experiments were conducted using the highest standards of humane care in accordance with the NIH Guide for the Care and Use of Laboratory Animals and were approved by the University of Cincinnati Institutional Animal Care and Use Committee. C57BL/6J mice used in these experiments were originally purchased from the Jackson Labs and thereafter maintained in our colony. C57BL/6J mice were housed in a pathogen-free animal facility under a standard 12-hour light/12-hour dark cycle with *ad libitum* water and chow. *Ahr^-/-^*mice crossed for 7 generations into a C57BL/6J background were initially purchased from Jackson labs and thereafter maintained to date in our laboratory into the C57BL/6J genetic background (for about 14 years). On gestation day (GD) 7.5 pregnant *Ahr*^+/+^ dams were treated by oral gavage with either corn oil (vehicle) or with TCDD (0.1 or 1 μg/kg of maternal weight) in corn oil vehicle, which was repeated on embryo days E9.5 and E11.5; *Ahr*^-/-^ pregnant dams remained untreated throughout gestation.

1. **RNA.seq analysis**

Following euthanasia, hearts were immediately harvested and rinsed with RNA-Later (Ambion). Each heart was microdissected into right atrium, left atrium, and ventricles. Individual samples were stored in 200 µL of RNA-Later at -80°C until RNA extraction. Total RNA was extracted with the RNeasy Mini Kit with Proteinase K and DNAse steps (QIAGEN).

All steps of library construction, cluster generation, and HiSeq (Illumina) sequencing were performed with biological triplicate samples by the Genomics Sequencing Core of the Department of Environmental Health, University of Cincinnati. Library construction was done with the TruSeq RNA sample preparation kit (Illumina) using 1 μg of total RNA, with RNA integrity number ≥ 7.0 as determined with an Agilent 2100 Bioanalyzer (Agilent Technologies) to purify poly-A–containing mRNA with oligo-dT–attached magnetic beads. The purified mRNA was enzymatically fragmented, with random hexamers primed for first and second strand cDNA synthesis, followed by purification using Agencourt AMPure XP beads (Beckman Coulter). Overhangs in the double-strand cDNA were blunt-ended by end repair and adenylated with a single A-nucleotide at the 3´ end to prevent self-ligation in the following ligation step. AMPure XP bead-purified fragments were ligated to sample-specific indexing adapters and enriched by 10 cycles of PCR using adapter-specific primers. A 1-μL aliquot of purified PCR product (from a total sequencing library of 30 μL) was analyzed in an Agilent bioanalyzer using a DNA 1000 chip to check DNA size (~ 260 bp) and yield. To quantify the library concentration for clustering, the library was diluted 1:100 in a buffer containing 10 mM Tris-HCl, pH 8.0, and 0.05% Tween 20, and analyzed by quantitative PCR (qPCR) with a KAPA Library Quantification kit (KapaBiosystems) using an ABI 9700HT real-time PCR machine (Applied Biosystems). Equal amounts of six individually indexed cDNA libraries were pooled for clustering in an Illumina cBot system flow cell at a concentration of 8 pM using Illumina’s TruSeq SR Cluster Kit v3, and sequenced for 50 cycles using a TruSeq SBS kit on the Illumina HiSeq system. Each sample generated approximately 30 million sequence reads. Sequence reads were demultiplexed and exported to fastq files using CASAVA 1.8 software (Illumina). The reads were then aligned to the reference genome (mm10) using TopHat aligner. The counts of reads aligning to each gene’s coding region were summarized using ShortRead and associated Bioconductor packages (GenomicFeatures, IRanges, GenomicRanges, Biostrings, Rsamtools) for manipulating and analysis of next-generation sequencing data and custom-written R programs.

Differential gene expression analysis between *Ahr*^+/+^ and *Ahr*^-/-^ hearts or between AHR ligand-exposed and naïve *Ahr*^+/+^ hearts was performed separately. Statistical analyses were performed to identify differentially expressed genes for each comparison using the negative-binomial model of read counts as implemented in the Bioconductor DESeq package. Significant genes were selected based on a false-discovery rate–adjusted p-value < 0.0001. RNA.seq data was further analyzed with the RNA.seq data using Ingenuity Pathway Analysis (IPA; Ingenuity® Systems, http://www.ingenuity.com).

**Supplemental Figures**


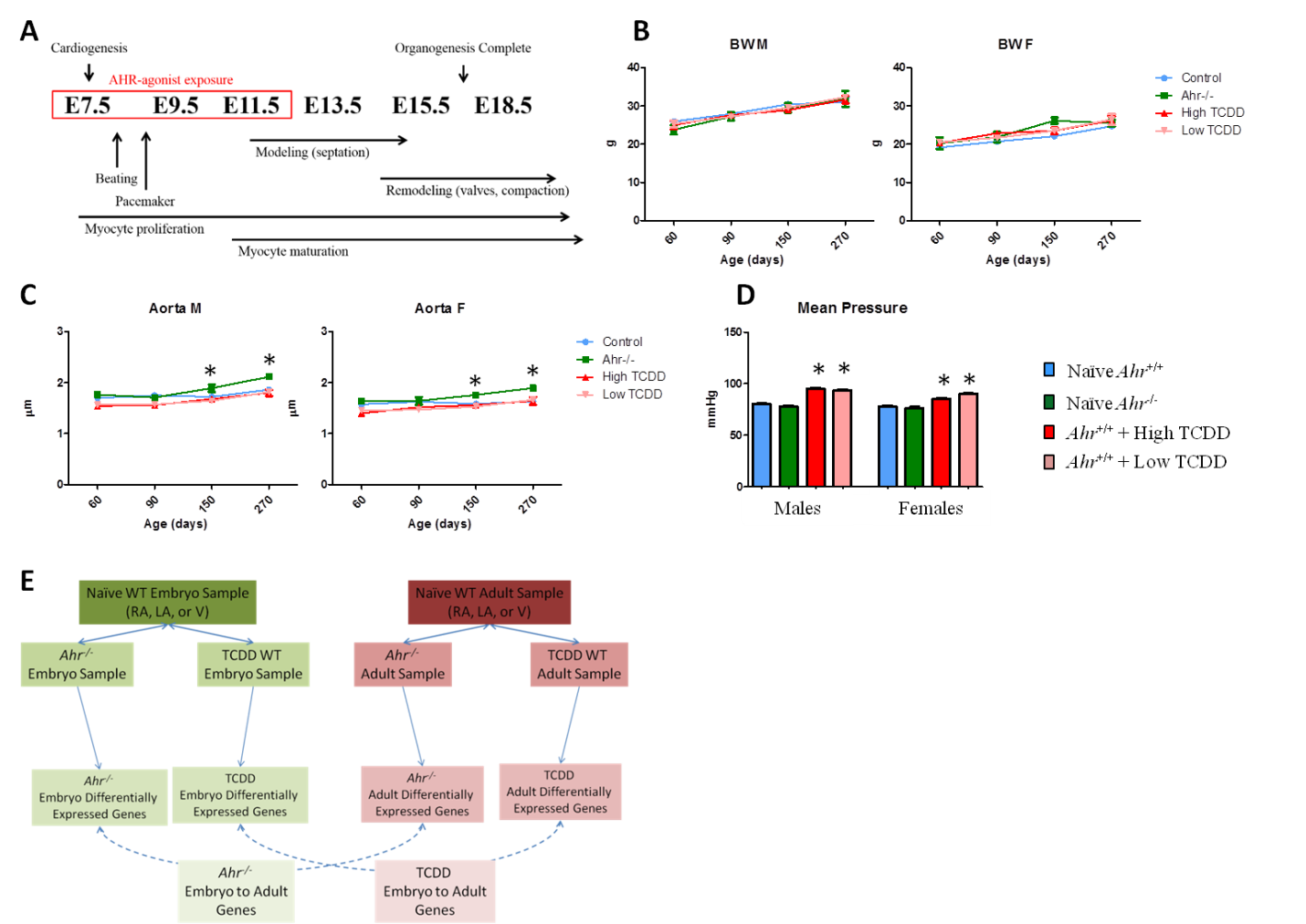


1. S1 Figures A-E - (A) Experimental design for gestational exposure to the prototypical AHR ligand (TCDD). (B) Body weight (grams) from naïve *Ahr^+/+^,* low- and high-dose ligand-exposed *Ahr^+/+^*, and *Ahr^-/-^* males and females at the indicated age (in days). Mean ± SEM. (C) Echocardiographic assessment of aorta diameter (µm) from naïve *Ahr^+/+^,* ligand-exposed *Ahr^+/+^*, and *Ahr^-/-^* males and females at the indicated age (in days). Mean ± SEM; * p≤0.05. (D) Mean blood pressure (mmHg) from naïve *Ahr^+/+^,* ligand-exposed *Ahr^+/+^*, and *Ahr^-/-^* males and females at 9 months (post natal day [PND] 270). Mean ± SEM; * p≤0.05. (E) RNA.seq data analysis design for adult heart transcriptome at PND 300 and comparison relative to embryo heart (i.e., persistent genes) transcriptome at either E13.5, E15.5, or E18.5.

**Supplemental Tables**

1. S1 Table A- Identity of genes differentially expressed in the embryo at either E13.5, E15.5, or E18.5 which persisted in the adult hearts at post natal day (PND) 300.

1. S1 Table B- Identity, function, targets, and fate of selected cardiovascular signaling pathways genes and proteins in cardiac hypertrophy and failure.

S1 Table C: Summary of cardiovascular findings related to developmental *Ahr* disruption in male and female mice. Arrow up = upregulated/increased, arrow down = downregulated/decreased, “↔ “ = not affected

|  | | **Male** | | **Female** | |
| --- | --- | --- | --- | --- | --- |
| **Ahr Disruption Condition** | | ***Ahr^-/-^*** | **TCDD** | ***Ahr^-/-^*** | **TCDD** |
| Molecular Findings  (affected networks) | Congenital Heart Anomaly | ↔ | ↔ | ↔ | 🡹 |
|  | Cardiac Homeostasis | ↔ | 🡻 | 🡻 | 🡻 |
|  | Cardiac Stress | ↔ | ↔ | ↔ | 🡹 |
|  | Mitochondrial Dysfunction | 🡹 | 🡹 | 🡹 | 🡹 |
|  | Energy Metabolism | ↔ | ↔ | ↔ | 🡻 |
| Heart Structure | Heart Weight | 🡹 | ↔ | 🡹 | 🡹 |
|  | LV Mass | 🡹 or ↔ | 🡹 or ↔ | 🡹 | ↔ |
|  | LV Volume | 🡹 | 🡹 or↔ | 🡹 | ↔ |
|  | Myofiber Size | ↔ | 🡹 | 🡻 | ↔ |
|  | Fibrosis | 🡹 | 🡹 | 🡹 | 🡹 |
| Heart Function | Heart Rate | ↔ | 🡹 | 🡻 | ↔ |
|  | Blood Pressure | ↔ | 🡹 | ↔ | 🡹 |
|  | Endurance | 🡻 | 🡻 | 🡻 | 🡻 or↔ |
| Mitochondria | Oxidative Phosphorylation | ↔ | ↔ | ↔ | 🡻 |
|  | Mitochondrial Abundance | ↔ | ↔ | ↔ | 🡹 |
